# Community awareness of genetic disorders associated with consanguineous marriage and acceptance of preventive screening, a cross-sectional study from Saudi Arabia

**DOI:** 10.3389/fgene.2026.1866894

**Published:** 2026-07-13

**Authors:** Abdulmajeed A. A. Sindi, Uthman Albakri, Sarah A. Alzahrani, Haneen M. Alqarni, Rimiyyah K. Alzahrani, Batol M. Albanghali, Mohammed A. Shanawaz, Basim A. Othman, Dhuha F. Alghamdi, Aryam A. Alghamdi, Ali Mahzari, Dina Marghani, Mohammad A. Albanghali

**Affiliations:** 1 Department of Basic Applied Medical Sciences, Faculty of Applied Medical Sciences, Al-Baha University, Al Baha, Saudi Arabia; 2 Department of Public Health, Faculty of Applied Medical Sciences, Al-Baha University, Al Baha, Saudi Arabia; 3 Department of Laboratory Medicine, Faculty of Applied Medical Sciences, Al-Baha University, Al Baha, Saudi Arabia; 4 Faculty of Medicine, Al-Baha University, Al Baha, Saudi Arabia; 5 Clinical Laboratory Science Department, Faculty of Applied Medical Sciences, Taibah University, Madina, Saudi Arabia

**Keywords:** autosomal recessive inheritance, consanguinity, genetic risk perception, inherited genetic disorders, Saudi Arabia

## Abstract

**Background:**

This study assessed community awareness, knowledge, and attitudes toward genetic disorders associated with consanguineous marriage and evaluated acceptance of premarital/genetic screening among adults in Al-Baha, Saudi Arabia.

**Methods:**

A descriptive cross-sectional study was conducted in late 2025 (October–November) among adults (≥18 years) residing in Al-Baha using a structured, self-administered electronic questionnaire distributed through social media, university channels, and community networks.

**Results:**

A total of 673 complete responses were analysed (mean age 32.67 ± 13.12 years; 50.4% female; 49.8% married). Consanguinity was common (tribal endogamy 42.5%; extended-family endogamy 40.0%), with 20.5% reporting first-cousin and 12.9% second-cousin marriage; parental and grandparental consanguinity were reported by 70.7% and 70.6%, respectively. Familial genetic/health burden was notable, particularly hereditary visual impairment (34.8%), thalassemia/hereditary anemia (25.7%), and hearing impairment/delayed speech (20.5%). Knowledge was generally good (84.4% recognized increased autosomal recessive risk; 89.3% endorsed genetic counselling benefits), but gaps persisted (33.1% did not know premarital screening includes SCD/thalassemia; only 42.1% correctly rejected the absence of a sensory-disorder link). Attitudes strongly supported prevention (premarital screening mandatory 93.9%), although within-family/tribe marriage remained widely endorsed (80.4%). Higher knowledge was associated with more favourable attitudes (OR = 2.25, p < 0.001); consanguinity with higher familial burden (OR = 2.45, p < 0.001); and familial burden with higher knowledge (OR = 1.65, p = 0.003) and more favourable attitudes (OR = 1.84, p < 0.001). A discordant profile of high preventive attitudes coexisting with acceptance of within-family or tribal marriage was observed in 51.9% of participants.

**Conclusion:**

Adults in Al-Baha demonstrated strong acceptance of preventive genetic services alongside generally good knowledge; however, persisting gaps, particularly related to awareness of specific service components and recognition of sensory-disorder links, support targeted community education and strengthened genetic counselling to optimize informed marital and reproductive decision-making.

## 1 Background

Consanguineous marriage (CM), defined as marriage between biologically related partners, remains common in many Arab populations and is strongly shaped by social, cultural, and kinship norms (Hamamy, 2012; Tadmouri et al., 2009). In Saudi Arabia, national data show a consistently high prevalence of consanguinity (56%), with first-cousin unions being the most frequent; importantly, prevalence varies by region, with Al-Baha historically among the lower-prevalence regions (42%) (El-Mouzan et al., 2007; El-Hazmi et al., 1995). More recent, region-focused work from Al-Baha continues to indicate that CM persists as a prominent marital pattern, reinforcing the need for locally grounded public-health assessment and response (Albanghali, 2023). From a public-health perspective, CM increases the probability that both partners carry the same pathogenic recessive variants, thereby increasing the risk of autosomal recessive disorders and contributing to preventable familial disease burden (Hamamy, 2012; Khayat et al., 2024). Saudi genomic and epidemiologic literature has repeatedly highlighted consanguinity as a key upstream driver of inherited disease patterns, emphasizing that risk communication and culturally appropriate prevention strategies are central to reducing long-term morbidity and healthcare costs (Khayat et al., 2024; Yousef et al., 2024).

In response to this public-health burden, Saudi Arabia has implemented a national preventive strategy through the Premarital Screening and Genetic Counselling (PMSGC)/“Healthy Marriage” program. The program was established by law in late 2003 and implemented in early 2004, initially focusing on hemoglobinopathies (notably sickle cell disease and thalassemias), with infectious disease screening later incorporated (e.g., HIV, HBV, HCV) (Ibrahim et al., 2013; Gosadi, 2019; Al Zuayr et al., 2024). National guidance documents emphasize prevention of hereditary and infectious disease transmission, structured counselling pathways, and standardized service delivery across regions (Ministry of Health, 2021). Evaluations of the program have demonstrated measurable public-health impact, including reductions in high-risk marriage certifications over time, supporting PMSGC as a cornerstone population-level intervention in Saudi Arabia (Memish and Saeedi, 2011; Alotaibi et al., 2025).

Despite this national infrastructure, the effectiveness of premarital services ultimately depends on community knowledge, perceived benefits and barriers, and willingness to act on results. Prior Saudi studies have documented variable public awareness and persistent social pressures that can limit behavioural change, including proceeding with marriage despite incompatible screening outcomes (Ibrahim et al., 2013; Al-Shroby et al., 2021; Arafah et al., 2021). Recent national and regional surveys similarly report that misconceptions and culturally mediated decision-making remain relevant, underscoring the need to strengthen education, counselling accessibility, and trust in genetic services (Yousef et al., 2024; Alwhaibi et al., 2024; Al Eissa et al., 2024). Accordingly, this study was designed to assess community awareness, knowledge, and attitudes toward genetic disorders associated with consanguineous marriage, and to evaluate acceptance of premarital and genetic screening programs among adults in Al-Baha.

## 2 Materials and methods

### 2.1 Study design and setting

A descriptive cross-sectional study was conducted between October and November 2025 to evaluate awareness, knowledge, and attitudes toward genetic disorders associated with consanguineous marriage among adults in the Al-Baha region, Saudi Arabia.

### 2.2 Ethics approval and consent to participate

This study was reviewed and approved by the Deanship of Scientific Research, Al-Baha University, Saudi Arabia (Approval No.: 1447-21-47114231-1). Electronic informed consent was obtained from all participants prior to enrolment using the online survey platform. The consent statement was presented on the first page of the survey, and participants were required to indicate their agreement (e.g., by selecting “I agree”) before accessing and completing the questionnaire. Participation was voluntary, and responses were collected anonymously.

### 2.3 Study population and eligibility criteria

The study included adults aged 18 years and older residing in the Al-Baha region. Both married and unmarried individuals were eligible to ensure community-level assessment of awareness and preventive attitudes. Individuals younger than 18 years, non-residents, and respondents with incomplete questionnaires were excluded from the analysis.

### 2.4 Sampling technique and sample size

Participants were recruited using a convenience sampling approach through an electronic questionnaire distributed via social media, university channels, and community networks. Sample size was calculated using the Raosoft calculator (Raosoft, Inc. Sample Size Calculator. Seattle, WA, USA), assuming a consanguinity prevalence of 41%, a 95% confidence level, and a 5% margin of error, yielding a minimum required sample of 384 participants. A total of 673 complete responses were included in the final analysis.

### 2.5 Study instrument

Data were collected using a structured, self-administered questionnaire developed to assess consanguinity patterns, familial genetic disease burden, and community awareness and attitudes toward genetic disorders and preventive screening in the Al-Baha region. The instrument was informed by relevant literature and adapted to the Saudi sociocultural context. It comprised five components: sociodemographic characteristics (7 items); family and community consanguinity patterns (5 items), including degree and frequency of relative marriage and parental consanguinity; health and genetic conditions (26 items) covering major autosomal recessive disorders, inborn errors of metabolism, sensory and neurodevelopmental conditions, and selected multifactorial diseases; knowledge and awareness of genetic disorders (9 items) assessed using true/false/do not know statements; attitudes toward premarital screening and genetic counselling (6 items) measured on a five-point Likert scale. The questionnaire was reviewed by subject-matter experts to ensure clarity and content validity prior to distribution, participation was voluntary, and no identifying information was collected.

### 2.6 Statistical analysis

Analyses were performed in IBM SPSS Statistics v21.0 (IBM Corp., Armonk, NY, USA). Categorical variables are presented as n (%) and continuous variables as mean ± SD or median (IQR). Pooled knowledge (0–9; items 5 and 8 reverse-coded) and attitude (6–30) scores were computed, internal consistency assessed by Cronbach’s α, and each score dichotomised at the sample median (cut-offs 6 and 25).

Bivariate associations were tested by Pearson’s chi-square with unadjusted odds ratios (OR) and 95% confidence intervals (CI). A sensitivity analysis applied post-stratification weights to the Saudi adult age × sex distribution (GASTAT 2022 Census), with weighted ORs from binomial generalised linear models. Outcomes across kinship strata (no relation/second cousin/first cousin; n = 377) were compared by chi-square and the Cochran–Armitage trend test. Independent predictors of high knowledge and high attitudes were identified by multivariable logistic regression adjusted for sex, age, education, income, marital status, residence, employment, parental consanguinity, and familial burden, with adjusted ORs (aOR) and 95% CIs reported. Item-level performance was stratified by age and education, and an attitude–behaviour discordance profile (high attitude with endorsement of within-family/tribe marriage) and its predictors were examined among participants with high attitudes. Tests were two-tailed with p < 0.05 considered significant; complete cases were used.

## 3 Results

### 3.1 Sociodemographic characteristics and patterns of consanguinity

A total of 673 adult residents of the Al-Baha region completed the questionnaire and were retained in the analytic sample. The mean age was 32.67 ± 13.12 years (median 28, interquartile range 21.5–43.0). Participants aged 18–25 years constituted the largest age stratum (n = 287, 42.6%), followed by 26–40 years (n = 201, 29.9%), 41–60 years (n = 165, 24.5%), and ≥61 years (n = 20, 3.0%). The sample was evenly distributed by sex (50.4% female, 49.6% male). Nearly half of participants were currently married (n = 335, 49.8%) and 46.1% were single. Most resided in the Eastern Governorates (44.9%) or Al-Baha City (43.2%). Educational attainment was predominantly bachelor’s level (49.8%) and 39.5% reported being unemployed.

Consanguinity was a prominent feature of the social environment. The most frequent predominant marriage pattern was tribal endogamy (n = 286, 42.5%) followed by extended-family endogamy (n = 269, 40.0%); exogamy was reported by 17.5% of participants. Relative marriages were perceived as sometimes (45.9%) or very common (44.3%) within the participants’ tribe or extended family. Regarding the current or most recent spousal relationship, 20.5% reported a first-cousin marriage and 12.9% a second-cousin marriage, while 22.6% reported no kinship and 44.0% were not married. Intergenerational consanguinity was high: 70.7% of participants reported parental consanguinity and 70.6% reported grandparental consanguinity (Table 1).

**TABLE 1:** Demographic characteristics and consanguinity patterns of the study population (N = 673).

​	Mean ± SD	Median (IQR)	​	​	​
Age (years)	32.67 ± 13.12	28 (21.5–43.0)	Characteristic	​	​
​	Frequency	%	​	Frequency	%
Age (years)			City/residence		
18-25	287	42.6	Al-Baha city	291	43.2
26-40	201	29.9	Coastal governorates	79	11.7
41-60	165	24.5	Eastern governorates	302	44.9
≥61	20	3	Education		
Sex			Primary	17	2.5
Female	339	50.4	Secondary	176	26.2
Male	334	49.6	Diploma	103	15.3
Marital status			Bachelor	335	49.8
Divorced	22	3.3	Postgraduate	42	6.2
Married	335	49.8	Employment		
Single	310	46.1	Employed (HS)	47	7
Widowed	6	0.9	Employed (non-HS)	213	31.6
Monthly family income			Student (HS)	60	8.9
<5k	136	20.2	Student (non-HS)	87	12.9
5k–9999	189	28.1	Unemployed	266	39.5
10k–14999	179	26.6	Type of relationship with current/most recent spouse		
≥15,000	169	25.1	No relation	152	22.6
Predominant marriage pattern in the family/social environment			First cousin	138	20.5
Exogamy	118	17.5	Second cousin	87	12.9
Extended family endogamy	269	40	Parental consanguinity		
Tribal endogamy	286	42.5	Yes	476	70.7
Perceived commonness of relative marriages within tribe/extended family			No	197	29.3
Never	11	1.6	Grandparental consanguinity		
Rare	55	8.2	Yes	475	70.6
Sometimes	309	45.9	No	198	29.4
Very common	298	44.3	​	​	​

Data are presented as mean ± standard deviation (SD), median (interquartile range [IQR]), or number (percentage). HS indicates Health Sciences. Monthly family income is reported in Saudi Riyals (SAR).

### 3.2 Self-reported familial burden of genetic disorders

Participants reported a substantial familial burden of hereditary and congenital conditions, providing important context for interpreting community awareness, attitudes, and acceptance of preventive screening. The most frequently endorsed family histories were hereditary visual impairment (34.8%), thalassemia/hereditary anemia (25.7%), hearing impairment or delayed speech (20.5%), sickle cell anemia (15.3%), G6PD deficiency (13.2%), and congenital heart defects (13.1%) (Table 2). Overall, 56.5% of participants reported at least one familial genetic or congenital condition, indicating widespread family-level exposure to inherited disorders in the population.

**TABLE 2:** Family history of genetic disorders among participants.

Genetic disorders	*n* (%)
Hereditary visual impairment	234 (34.8)
Thalassemia (mediterranean anemia)	173 (25.7)
Hearing impairment/delayed speech	138 (20.5)
Sickle cell anemia (sickle cell disease)	103 (15.3)
G6PD deficiency (favism)	89 (13.2)
Congenital heart defects (e.g., septal defects)	88 (13.1)
Hereditary milk intolerance/galactosemia	76 (11.3)
Hereditary renal failure	70 (10.4)
Hereditary kidney stones (cystinuria)	67 (10.0)
Developmental delay or motor disability	59 (8.8)
Cystic fibrosis (hereditary lung disease)	46 (6.8)
Spinal muscular atrophy/muscular dystrophy	45 (6.7)
Organic acidemia (propionic acidemia group)	26 (3.9)
Severe neonatal jaundice/kernicterus	20 (3.0)
Maple syrup urine disease (MSUD)	19 (2.8)
Urea cycle disorder/hyperammonemia (argininosuccinic aciduria group)	10 (1.5)
Glycogen storage disease (GSD)/metabolic disorder (GSD type I)	10 (1.5)
Urea cycle disorder (other subtypes)	9 (1.3)
Elevated homocysteine (hyperhomocysteinemia)	8 (1.2)
Organic acidemia/MMA deficiency (methylmalonic acidemia group)	7 (1.0)
3-MCC enzyme deficiency	6 (0.9)
Long-chain fatty acid oxidation defect (VLCAD deficiency)	6 (0.9)
Medium-chain fatty acid oxidation defect (MCAD deficiency)	5 (0.7)
Beta-ketothiolase deficiency	3 (0.4)
Beta-glucuronidase enzyme deficiency	2 (0.3)
Butyryl-CoA dehydrogenase deficiency	1 (0.1)
≥1 familial condition (any of the above)	380 (56.5)

### 3.3 Knowledge and awareness of genetic disorders associated with consanguineous marriage

Internal consistency of the 9-item knowledge scale was acceptable (Cronbach’s α = 0.67); corrected item–total correlations ranged from 0.25 to 0.46, with the two negatively worded items among the lowest (item 5: r = 0.27; item 8: r = 0.25), consistent with the greater interpretive difficulty of reverse-worded statements. Overall, participants showed good knowledge of the genetic implications of consanguinity and of preventive measures (Table 3). Most respondents correctly identified that consanguineous marriage increases the probability of autosomal recessive disorders (84.4%), that repeated intra-family marriage increases hereditary risk (78.3%), and that genetic counselling reduces recurrence within families (89.3%). Awareness was also high regarding community-based genetic studies (90.6%), the potential expansion of premarital screening (82.2%), and the role of newborn screening in early detection of inherited metabolic disorders (74.6%). Nevertheless, gaps persisted: only 66.1% correctly reported that the Saudi premarital screening programme includes sickle cell disease and thalassemia (33.1% selected ‘do not know’), only 42.1% correctly rejected the statement that consanguinity has no association with hearing or vision disorders, and only 59.7% correctly rejected the statement that newborn screening is not fully implemented in Saudi Arabia. The pooled knowledge score (cut-off 6/9) classified 70.7% of participants as having high knowledge.

**TABLE 3:** Participants’ knowledge regarding consanguinity and genetic screening programs.

No	Items	True *n* (%)	False *n* (%)	Don’t know *n* (%)
1	Consanguineous marriage increases the probability of autosomal recessive disorders	568 (84.4)	21 (3.1)	84 (12.5)
2	Premarital screening in Saudi Arabia includes sickle cell anemia and thalassemia	445 (66.1)	5 (0.7)	223 (33.1)
3	Premarital screening can be expanded to identify carriers of additional hereditary disorders	553 (82.2)	5 (0.7)	115 (17.1)
4	Newborn screening detects some inherited metabolic disorders early	502 (74.6)	9 (1.3)	162 (24.1)
5	Newborn screening is not fully implemented in Saudi Arabia^†^	61 (9.1)	402 (59.7)	210 (31.2)
6	Repeated marriage within the same family increases the risk of hereditary disorders	527 (78.3)	28 (4.2)	118 (17.5)
7	Genetic counselling reduces the recurrence of hereditary diseases in families	601 (89.3)	6 (0.9)	66 (9.8)
8	Consanguinity has no association with hereditary hearing or vision disorders^†^	142 (21.1)	283 (42.1)	248 (36.8)
9	Community-based genetic studies help prevent hereditary diseases	610 (90.6)	5 (0.7)	58 (8.6)

^†^ Responses reflect participants’ selection of True, False, or Don’t know for each statement. For negatively worded items, the scientifically correct response is False. Specifically, for Item 5 (“Newborn screening is not fully implemented in Saudi Arabia”) and Item 8 (“Consanguinity has no association with hearing or vision disorders”), selecting False indicates correct knowledge, whereas selecting True indicates an incorrect belief; Don’t know indicates uncertainty.

### 3.4 Attitudes toward premarital screening and genetic counselling

**FIGURE 1:**
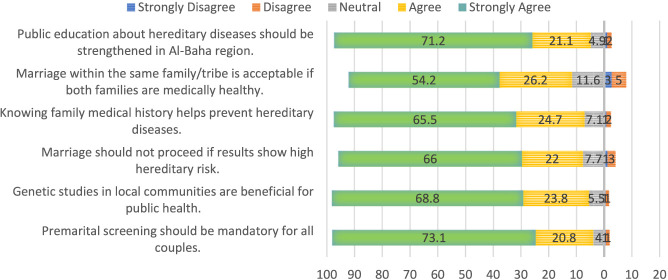
Distribution of participants’ attitudes toward genetic and premarital screening in Al-Baha (N = 673).

Internal consistency of the 6-item attitude scale was excellent (Cronbach’s α = 0.90; all corrected item–total correlations ≥0.55). Participants demonstrated strongly favourable attitudes toward preventive genetic measures and screening. The proportion who agreed or strongly agreed was 93.9% for mandatory premarital screening, 92.7% for the public-health value of community-based genetic studies, 92.4% for strengthening public education about hereditary diseases in Al-Baha, 90.3% for the role of family medical history in disease prevention, and 88.0% for not proceeding with marriage when screening indicates high hereditary risk. Comparatively, agreement was lower—though still substantial—for the statement that marriage within the same family or tribe is acceptable if both families are medically healthy (80.4%; Figure 1). The pooled attitude score, dichotomised at the median (cut-off 25/30), classified 61.1% of participants as having high attitudes and 38.9% as having low attitudes.

### 3.5 Associations between knowledge, consanguinity, familial burden, and preventive attitudes

Bivariate analyses identified several significant relationships among consanguinity status, familial genetic/health condition burden, knowledge, and preventive attitudes (Table 4). Higher knowledge was significantly associated with more favourable preventive attitudes; participants with high knowledge were more likely to have high attitude scores than those with low knowledge (OR = 2.25, 95% CI: 1.60–3.16; p < 0.001). Consanguinity was strongly associated with reported familial genetic/health conditions, with participants in consanguineous marriages more frequently reporting ≥1 hereditary/genetic condition in the family (OR = 2.45, 95% CI: 1.61–3.75; p < 0.001). Familial genetic/health condition burden was also associated with both higher knowledge (OR = 1.65, 95% CI: 1.18–2.30; p = 0.003) and more favourable attitudes toward preventive screening (OR = 1.84, 95% CI: 1.34–2.52; p < 0.001). In contrast, consanguinity was not significantly associated with knowledge category (OR = 0.72, 95% CI: 0.46–1.13; p = 0.150) or attitude category (OR = 1.27, 95% CI: 0.84–1.93; p = 0.260).

**TABLE 4:** Pairwise associations between consanguinity, familial genetic disease burden, knowledge, and attitudes toward preventive screening among participants.

Characteristic	​	Consanguinity marriage			Genetic/Health conditions			Pooled knowledge score			Pooled attitude score		
		No	Yes	p-value	No	Yes	p-value	Low	High	p-value	Low	High	p-value
Consanguinity marriage	No	​	​	​	95 (62.5)	57 (37.5)	<0.001	42 (35.0)	110 (42.8)	0.150	69 (45.4)	83 (54.6)	0.260
	Yes	​	​	​	91 (40.4)	134 (59.6)		78 (65)	147 (57.2)		89 (39.6)	136 (60.4)	
Genetic/Health conditions	No	​	​	​	​	​	​	103 (52.3)	190 (39.9)	0.003	138 (52.7)	155 (37.7)	<0.001
	Yes	​	​	​	​	​	​	94 (47.7)	286 (60.1)		124 (47.3)	256 (62.3)	
Pooled knowledge score	Low	​	​	​	​	​	​	​	​	​	104 (39.7)	93 (22.6)	<0.001
	High	​	​	​	​	​	​	​	​	​	158 (60.3)	318 (77.4)	
Pooled attitude score	Low	​	​	​	​	​	​	​	​	​	​	​	​
	High	​	​	​	​	​	​	​	​	​	​	​	​

Values are presented as n (%), with percentages calculated within the column category for each pairwise comparison. P-values are from Pearson’s chi-square test (two-sided). Genetic/health conditions indicator (family history/presence of ≥1 reported hereditary/genetic condition: yes/no); Pooled knowledge score category: low/high; cut-off = 6; Pooled attitude score category: low/high; cut-off = 25.

### 3.6 Sensitivity and subgroup analyses

Post-stratification weighting was applied to assess the influence of non-probability sampling using the Saudi adult age × sex distribution from GASTAT 2022 Census tabulations. The sample over-represented adults aged 18–25 years and under-represented adults aged ≥61 years, while the sex distribution was broadly balanced (Table 5). Weighted estimates were like unweighted estimates for consanguineous current or most recent marriage among ever-married participants, ≥1 familial condition, high knowledge, and high preventive attitudes: 61.3%, 54.5%, 69.4%, and 60.7%, respectively, compared with 59.7%, 56.5%, 70.7%, and 61.1% unweighted. The four primary associations remained statistically significant after weighting: high knowledge with high attitudes (OR = 2.00, 95% CI: 1.43–2.79; p < 0.001), consanguinity with familial conditions (OR = 2.68, 95% CI: 1.81–3.95; p < 0.001), familial conditions with high knowledge (OR = 1.49, 95% CI: 1.07–2.07; p = 0.018), and familial conditions with high attitudes (OR = 1.93, 95% CI: 1.41–2.64; p < 0.001). Among ever-married participants with a defined spousal relationship, familial conditions increased with kinship closeness: 37.5% in unrelated marriages, 49.4% in second-cousin marriages, and 65.9% in first-cousin marriages (Pearson χ^2^ p < 0.001; trend p < 0.001; Table 6). No significant trend was observed for high knowledge or high attitudes.

**TABLE 5:** Achieved sample distribution compared with the Saudi adult (≥18 years) reference distribution.

Stratum	Sample, n (%)	Reference (%)	Post-stratification weight
Age group			
18–25	287 (42.6)	24.5	0.57
26–40	201 (29.9)	37.8	1.26
41–60	165 (24.5)	27.7	1.13
≥61	20 (3.0)	10.0	3.33
Sex			
Female	339 (50.4)	≈50	≈1.00
Male	334 (49.6)	≈50	≈1.00

Reference percentages were derived from publicly available GASTAT 2022 Census tabulations for the Saudi national adult population, used as a proxy in the absence of a directly published Al-Baha-specific adult age × sex breakdown. Weights are presented as approximate marginal ratios for transparency; final case weights were computed from the full age × sex joint distribution. After trimming at the 99th percentile and normalisation to mean 1, weights ranged from 0.48 to 1.86.

**TABLE 6:** Outcomes by kinship closeness of current or most recent spousal relationship (ever-married participants, n = 377).

Outcome	No relation (n = 152)	Second cousin (n = 87)	First cousin (n = 138)	χ^2^ p	Trend p
≥1 familial condition, n (%)	57 (37.5)	43 (49.4)	91 (65.9)	<0.001	<0.001
High knowledge (≥6/9), n (%)	110 (72.4)	56 (64.4)	91 (65.9)	0.345	0.233
High attitude (≥25/30), n (%)	83 (54.6)	51 (58.6)	85 (61.6)	0.481	0.227

χ^2^ p, two-sided Pearson chi-square test across the three groups. Trend p, Cochran–Armitage test for linear trend across kinship closeness ordered as no relation < second cousin < first cousin.

### 3.7 Multivariable predictors and item-level knowledge gaps

In adjusted logistic regression, tertiary education and reporting ≥1 familial condition were independently associated with high knowledge. Tertiary education was associated with higher odds of high knowledge (aOR = 1.58, 95% CI: 1.06–2.37; p = 0.025), as was reporting ≥1 familial condition (aOR = 1.54, 95% CI: 1.09–2.18; p = 0.014). High preventive attitudes were independently associated with tertiary education (aOR = 1.54, 95% CI: 1.05–2.25; p = 0.026), parental consanguinity (aOR = 1.44, 95% CI: 1.02–2.04; p = 0.039), and reporting ≥1 familial condition (aOR = 1.70, 95% CI: 1.23–2.35; p = 0.001). Other covariates were not independently associated with either outcome (Table 7).

**TABLE 7:** Multivariable logistic regression of predictors of high knowledge and high preventive attitudes (N = 673).

Predictor (reference)	High knowledge aOR (95% CI), p	High attitude aOR (95% CI), p
Female (vs. male)	1.19 (0.81–1.74), 0.378	1.18 (0.82–1.68), 0.371
Age 26–40 (vs. 18–25)	0.67 (0.38–1.18), 0.171	0.73 (0.43–1.24), 0.244
Age 41–60 (vs. 18–25)	0.59 (0.31–1.12), 0.108	0.77 (0.42–1.43), 0.413
Age ≥61 (vs. 18–25)	0.54 (0.19–1.53), 0.245	1.20 (0.42–3.47), 0.734
Tertiary education (vs. ≤secondary)	1.58 (1.06–2.37), 0.025*	1.54 (1.05–2.25), 0.026*
Family income ≥10,000 SAR (vs. <10,000)	1.28 (0.90–1.81), 0.171	1.06 (0.77–1.47), 0.722
Married (vs. not married)	1.11 (0.66–1.87), 0.701	0.98 (0.60–1.62), 0.947
Al-Baha city residence (vs. other)	0.85 (0.60–1.20), 0.342	1.11 (0.80–1.55), 0.515
Employed (vs. not employed)	1.02 (0.64–1.61), 0.946	1.25 (0.81–1.93), 0.315
Parental consanguinity (vs. no)	0.89 (0.61–1.30), 0.543	1.44 (1.02–2.04), 0.039*
≥1 familial condition (vs. none)	1.54 (1.09–2.18), 0.014*	1.70 (1.23–2.35), 0.001*

aOR, adjusted odds ratio; CI, confidence interval. Models adjusted for all variables shown. *p < 0.05.

Item-level analysis showed that the main knowledge gaps concerned the link between consanguinity and hereditary hearing or vision disorders, newborn-screening implementation in Saudi Arabia, and the scope of the national premarital screening programme (Table 8). Correct recognition of autosomal recessive risk declined with age, from 87.5% among participants aged 18–25 years to 55.0% among those aged ≥61 years (p < 0.001). Tertiary education was associated with higher correct response rates for several key items, including autosomal recessive risk, repeated intra-family marriage risk, genetic counselling, and community-based genetic studies.

**TABLE 8:** Item-level knowledge performance by age group and education (N = 673).

Item (correct response)	Overall %	18%–25%	26%–40%	41%–60%	≥61%	p (age)	≤Sec %	Tertiary %	p (educ.)
1. Consanguinity ↑ AR risk (true)	84.4	87.5	87.1	79.4	55.0	<0.001	78.2	86.9	0.007
2. PMS includes SCD/thal (true)	66.1	68.3	65.2	64.2	60.0	0.734	61.7	67.9	0.144
3. PMS can be expanded (true)	82.2	79.8	80.1	87.9	90.0	0.102	77.7	84.0	0.072
4. NBS detects metabolic disorders early (true)	74.6	76.0	76.1	69.7	80.0	0.403	69.4	76.7	0.064
5. NBS not fully implemented in SA (False)†	59.7	62.4	66.2	49.1	45.0	0.003	61.7	59.0	0.576
6. Repeated intra-family marriage ↑ risk (true)	78.3	78.0	77.6	81.8	60.0	0.157	71.0	81.2	0.005
7. Genetic counselling reduces recurrence (true)	89.3	86.8	92.5	88.5	100.0	0.084	83.4	91.7	0.003
8. Consanguinity has no link to hearing/vision (False)†	42.1	44.9	41.3	38.8	35.0	0.539	39.9	42.9	0.528
9. Community-based genetic studies help prevent (true)	90.6	88.5	91.5	92.7	95.0	0.386	85.5	92.7	0.006

† Negatively worded items; the correct response is False. AR, autosomal recessive; PMS, premarital screening; NBS, newborn screening; SCD, sickle cell disease; thal = thalassemia. Percentages reflect the proportion correct within each stratum. Education dichotomised as Secondary versus Tertiary (Diploma/Bachelor/Postgraduate). Chi-square p-values are two-sided.

### 3.8 Attitude–behaviour discordance

Although preventive attitudes were favourable, acceptance of endogamy remained common. Overall, 80.4% agreed or strongly agreed that marriage within the same family or tribe is acceptable when both families are medically healthy. A discordant profile, defined as high preventive attitudes combined with acceptance of within-family or tribal marriage, was observed in 349 participants, or 51.9% of the sample. Only 62 participants, or 9.2%, had high preventive attitudes while rejecting within-family or tribal marriage. Among participants with high preventive attitudes, parental consanguinity showed a borderline adjusted association with this discordant profile (aOR = 1.66, 95% CI: 0.91–3.03; p = 0.102). This finding suggests that favourable attitudes toward screening coexist with continued cultural acceptance of endogamy.

## 4 Discussion

This study provides community-level evidence from Al-Baha on patterns of consanguinity, perceived familial genetic/health burden, and acceptance of preventive genetic services. Consanguineous marriage remains a deeply rooted marital practice in many Arab societies, and its health relevance is largely mediated through increased homozygosity and the expression of autosomal recessive disorders (Hamamy, 2012; Hamamy et al., 2011). Nationally, Saudi Arabia has consistently reported high consanguinity prevalence with marked regional variation (El-Mouzan et al., 2007). In Al-Baha, recent regional data similarly indicate that consanguinity persists and remains epidemiologically relevant (Albanghali, 2023). In our sample, the predominance of tribal and extended-family endogamy, together with frequent reports of intergenerational consanguinity, indicates that kin marriage remains a common and culturally relevant practice in the region.

Participants reported a substantial familial burden of hereditary and congenital conditions, with visual impairment, hemoglobinopathies, and hearing impairment/delayed speech among the most frequently endorsed conditions. Consistent with this autozygosity-based mechanism, we observed a clear monotonic gradient in self-reported familial burden across kinship strata (37.5% in unrelated, 49.4% in second-cousin, and 65.9% in first-cousin marriages; trend p < 0.001), with no parallel increase in knowledge or preventive attitudes, indicating that couples in closer-kin marriages carry the greatest reported risk but are not yet better informed or more preventively oriented than couples in unrelated marriages (Hamamy, 2012; Hamamy et al., 2011). While self-reported family history cannot establish clinical diagnoses, the observed pattern is broadly consistent with the established relationship between consanguinity and increased occurrence of recessive and congenital conditions in highly endogamous populations (Hamamy, 2012; Hamamy et al., 2011). Importantly, our inferential findings showed that consanguinity was associated with higher odds of reporting ≥1 familial genetic/health condition, supporting the plausibility of cumulative familial burden in communities where endogamy is common. In addition, familial burden was associated with higher knowledge and more favourable preventive attitudes, suggesting that family experience may reinforce awareness and acceptance of preventive services.

Clinical genomic studies of consanguineous Saudi cohorts have demonstrated that autosomal recessive disorders dominate the Mendelian disease burden in the Kingdom, with a substantial proportion of recessive mutations identified through diagnostic sequencing being founder variants (Monies et al., 2017), and that population-based carrier-frequency estimation places the disease burden at approximately seven per 1,000 children born to first-cousin parents (Abouelhoda et al., 2016). Tribe- and region-specific variant cataloguing within the Saudi Human Genome Program (Alkuraya, 2014) supports the case for expanded carrier-screening panels tailored to specific Saudi tribes and regions; the kinship-burden gradient observed in our Al-Baha sample reinforces this case at the community level, and aligns the genomic-prevention agenda with the recessive conditions most frequently reported by Al-Baha families (visual impairment, hemoglobinopathies, sensory–neurodevelopmental conditions, and inborn errors of metabolism).

Knowledge items suggested generally strong awareness of genetic risk and the role of counselling; however, gaps persisted in service specific knowledge and outcome specific understanding, particularly regarding the association between consanguinity and hearing or vision disorders. This distinction between general genetic awareness and service-specific knowledge is programmatically important because Saudi Arabia’s preventive strategy relies heavily on structured premarital screening and genetic counselling (Ministry of Health, 2021). Multivariable analysis confirmed that tertiary education and direct family experience of a hereditary condition were the only independent predictors of high knowledge, identifying adults with lower educational attainment and without familial exposure as priority audiences for community education.

Prior Saudi studies have similarly reported that attitudes toward premarital screening may be favourable even when knowledge is incomplete, and that service experience and counselling quality can influence understanding and decision-making (Ibrahim et al., 2013). Consistent with this, our data showed that higher knowledge was associated with more favourable preventive attitudes, indicating that targeted education may further strengthen acceptance and informed decision making.

Attitudinal findings were strongly supportive of preventive measures (Figure 1), aligning with prior national work demonstrating high perceived value of premarital screening and counselling (Ibrahim et al., 2013; Al-Shroby et al., 2021). Notably, this support coexisted with continued endorsement of within-family or tribal marriage, highlighting a potential gap between preventive acceptance and marital practice. In this study, the gap was directly quantified: 51.9% of participants combined high preventive attitudes with endorsement of within-family or tribal marriage, whereas only 9.2% combined high attitudes with rejection of endogamy. Nevertheless, substantial endorsement of within-family/tribe marriage despite “medical health” reflects the persistence of social norms that may limit behavioural change even when preventive services are accepted in principle. Similar “attitude–behaviour” gaps where individuals proceed with marriage despite risk have been reported in Saudi premarital screening literature, underscoring the need for culturally sensitive counselling and risk communication that engages couples and families (Ibrahim et al., 2013; Al-Shroby et al., 2021). In relation to newborn screening, most participants correctly rejected the statement that newborn screening is not fully implemented; however, a substantial proportion selected “do not know,” indicating uncertainty regarding the current scope and implementation of national services beyond premarital screening. Our findings should be interpreted against Saudi Arabia’s layered preventive architecture. The PMSGC programme has demonstrated measurable reductions in at-risk marriage certifications over time (Memish and Saeedi, 2011), and is now complemented within Vision 2030 by digital health platforms such as Mawid and Sehhaty that have expanded service access but have not yet been systematically paired with SBCC content tailored to highly endogamous regions. The persistent endorsement of within-family or tribal marriage despite high acceptance of screening suggests that current SBCC efforts do not fully engage the kinship and tribal structures that shape marital decisions in Al-Baha; embedding genetic-risk literacy into imam- and community-leader-mediated dialogue, alongside digital channels, represents a culturally congruent extension of existing policy.

Findings support strengthening community education and counselling in Al-Baha, with emphasis on clarifying the scope and purpose of premarital screening services provided through the Saudi Ministry of Health and improving genetic risk communication in the context of endogamy (Ministry of Health, 2021). Evidence from the national premarital program indicates that sustained screening and counselling can reduce at-risk marriage certifications over time (Memish and Saeedi, 2011), suggesting that enhancing local awareness and counselling quality may further improve preventive impact.

The cross-sectional design limits causal inference. The electronic convenience sampling approach may introduce selection bias, and family history reporting may be affected by recall and diagnostic uncertainty. Additionally, dichotomizing pooled scores may reduce variability. These limitations should be considered when interpreting effect sizes and generalizability. The questionnaire did not capture participants’ sources of information on genetic disorders or premarital screening (e.g., Ministry of Health channels, primary care, school curricula, social media, family, or religious authorities), which limits attribution of observed knowledge patterns to specific communication channels; this should be addressed in subsequent regional surveys. Reassuringly, all four primary associations remained statistically significant after post-stratification weighting to the Saudi adult age × sex distribution, supporting the robustness of the inferential conclusions to the demographic profile of the achieved sample.

## 5 Conclusion

Consanguinity remains common and intergenerationally entrenched in Al-Baha, and the burden of self-reported hereditary conditions increased steadily with kinship closeness. Participants showed generally good awareness of consanguinity-related risk and strong support for preventive screening, yet specific gaps persisted, most notably around the consanguinity–sensory-disorder link, newborn-screening implementation, and the scope of the national programme. Crucially, over half of the sample combined favourable screening attitudes with continued acceptance of within-family or tribal marriage, indicating that improving knowledge alone will not change marital practice. Three priorities follow: focused education on the weakest knowledge areas; communication aimed at adults with lower education, older adults, and families without prior experience of hereditary disease; and stronger regional counselling capacity, including teleogenetic links to tertiary centres and tribe-tailored expanded carrier screening. Together, these measures would help convert the favourable preventive climate observed here into real change in marital and reproductive decision-making across Al-Baha and similar Saudi settings.

## Data Availability

The original contributions presented in the study are included in the article/Supplementary Material, further inquiries can be directed to the corresponding author.
