# Supplementary material for: Community awareness of genetic disorders associated with consanguineous marriage and acceptance of preventive screening, a cross-sectional study from Saudi Arabia
Source: Front Genet. 2026 Jul 13;17:1866894. doi: 10.3389/fgene.2026.1866894 (PMC13401984; doi:10.3389/fgene.2026.1866894)
Supplement: Supplementary file 1 [file Supplementaryfile1.docx]

**Supplementary: Questionnaire**

**Section A. Sociodemographic Characteristics (7 items)**

| **Item** | **Question** | **Response options** |
| --- | --- | --- |
| A1 | Age in completed years | Open numeric response: ______ |
| A2 | Sex | ☐ Female ☐ Male |
| A3 | Marital status | ☐ Single ☐ Married ☐ Divorced ☐ Widowed |
| A4 | Place of residence within the Al-Baha region | ☐ Al-Baha City ☐ Coastal Governorates ☐ Eastern Governorates |
| A5 | Highest level of education completed | ☐ Primary ☐ Secondary ☐ Diploma ☐ Bachelor ☐ Postgraduate |
| A6 | Current occupation | ☐ Employed in a health-sciences field ☐ Employed in a non-health-sciences field ☐ Student in a health-sciences field ☐ Student in a non-health-sciences field ☐ Unemployed |
| A7 | Monthly family income (Saudi Riyals, SAR) | ☐ Less than 5,000 ☐ 5,000–9,999 ☐ 10,000–14,999 ☐ 15,000 or more |

**Section B. Family and Community Consanguinity Patterns (5 items)**

| **Item** | **Question** | **Response options** |
| --- | --- | --- |
| B1 | What is the predominant marriage pattern in your family or social environment? | ☐ Exogamy (outside the family and tribe) ☐ Extended-family endogamy ☐ Tribal endogamy |
| B2 | How common are relative marriages within your tribe or extended family? | ☐ Never ☐ Rare ☐ Sometimes ☐ Very common |
| B3 | Type of relationship with your current or most recent spouse | ☐ Not applicable (never married) ☐ No biological relation ☐ First cousin ☐ Second cousin |
| B4 | Were your parents married within the same family or tribe? | ☐ Yes ☐ No If Yes, please specify the relationship: ______ |
| B5 | Were your grandparents (on either side) married within the same family or tribe? | ☐ Yes ☐ No If Yes, please specify the relationship: ______ |

*Items B4 and B5 include conditional open-ended follow-up sub-items, collected for descriptive purposes.*

**Section C. Family History of Genetic and Congenital Conditions (26 items)**

For each condition listed below, please indicate whether any biological relative within your family has been diagnosed. Response options for every item: ☐ Yes ☐ No.

| **Item** | **Condition** | **Response** |
| --- | --- | --- |
| C1 | Sickle cell anemia (sickle cell disease) | ☐ Yes ☐ No |
| C2 | Thalassemia / hereditary anemia (Mediterranean anemia) | ☐ Yes ☐ No |
| C3 | G6PD deficiency (favism) | ☐ Yes ☐ No |
| C4 | Severe neonatal jaundice / kernicterus | ☐ Yes ☐ No |
| C5 | Maple syrup urine disease (MSUD) | ☐ Yes ☐ No |
| C6 | Elevated homocysteine (hyperhomocysteinemia) | ☐ Yes ☐ No |
| C7 | Urea cycle disorder / hyperammonemia (argininosuccinic aciduria group) | ☐ Yes ☐ No |
| C8 | Hereditary kidney stones (cystinuria) | ☐ Yes ☐ No |
| C9 | Organic acidemia (propionic acidemia group) | ☐ Yes ☐ No |
| C10 | Organic acidemia / methylmalonic acidemia (MMA deficiency) | ☐ Yes ☐ No |
| C11 | 3-MCC enzyme deficiency | ☐ Yes ☐ No |
| C12 | Butyryl-CoA dehydrogenase deficiency | ☐ Yes ☐ No |
| C13 | Glycogen storage disease (GSD), type I | ☐ Yes ☐ No |
| C14 | Urea cycle disorder (other subtypes) | ☐ Yes ☐ No |
| C15 | Beta-glucuronidase enzyme deficiency | ☐ Yes ☐ No |
| C16 | Medium-chain fatty acid oxidation defect (MCAD deficiency) | ☐ Yes ☐ No |
| C17 | Long-chain fatty acid oxidation defect (VLCAD deficiency) | ☐ Yes ☐ No |
| C18 | Beta-ketothiolase deficiency | ☐ Yes ☐ No |
| C19 | Hereditary milk intolerance / galactosemia | ☐ Yes ☐ No |
| C20 | Congenital heart defects (e.g., septal defects) | ☐ Yes ☐ No |
| C21 | Hearing impairment / delayed speech | ☐ Yes ☐ No |
| C22 | Spinal muscular atrophy (SMA) / muscular dystrophy | ☐ Yes ☐ No |
| C23 | Hereditary renal failure | ☐ Yes ☐ No |
| C24 | Hereditary visual impairment | ☐ Yes ☐ No |
| C25 | Developmental delay or motor disability | ☐ Yes ☐ No |
| C26 | Cystic fibrosis (hereditary lung disease) | ☐ Yes ☐ No |

*Section C items follow the original survey order as administered. Frequencies are reported in Table 2 of the main manuscript (ordered by descending prevalence).*

**Section D. Knowledge and Awareness of Genetic Disorders (9 items)**

For each statement, please indicate whether you believe it is True, False, or whether you are not sure. Response options for every item: ☐ True ☐ False ☐ Don't know.

| **Item** | **Statement** | **Correct response** |
| --- | --- | --- |
| D1 | Consanguineous marriage increases the probability of autosomal recessive disorders. | True |
| D2 | Premarital screening in Saudi Arabia includes sickle cell anemia and thalassemia. | True |
| D3 | Premarital screening can be expanded to identify carriers of additional hereditary disorders. | True |
| D4 | Newborn screening detects some inherited metabolic disorders early. | True |
| D5 | Newborn screening is not fully implemented in Saudi Arabia. | False † |
| D6 | Repeated marriage within the same family increases the risk of hereditary disorders. | True |
| D7 | Genetic counselling reduces the recurrence of hereditary diseases in families. | True |
| D8 | Consanguinity has no association with hereditary hearing or vision disorders. | False † |
| D9 | Community-based genetic studies help prevent hereditary diseases. | True |

*† Negatively worded items (D5 and D8); the correct response is False. These items are reverse-coded when computing the pooled knowledge score. Response options actually shown to participants for every item were: True / False / Don't know. Item D5 and D8 wording is identical to that reported in Table 3 of the main manuscript.*

**Section E. Attitudes Toward Premarital Screening and Genetic Counselling (6 items)**

For each statement, please indicate your level of agreement on the five-point Likert scale shown below.

| **Coding** | | **Response anchor** | |
| --- | --- | --- | --- |
| 1 | | Strongly Disagree | |
| 2 | | Disagree | |
| 3 | | Neutral | |
| 4 | | Agree | |
| 5 | | Strongly Agree | |
| **Item** | **Statement** | | **Response options** |
| E1 | Premarital screening should be mandatory for all couples. | | 1 ☐ 2 ☐ 3 ☐ 4 ☐ 5 ☐ |
| E2 | Genetic studies in local communities are beneficial for public health. | | 1 ☐ 2 ☐ 3 ☐ 4 ☐ 5 ☐ |
| E3 | Marriage should not proceed if screening results show high hereditary risk. | | 1 ☐ 2 ☐ 3 ☐ 4 ☐ 5 ☐ |
| E4 | Knowing family medical history helps prevent hereditary diseases. | | 1 ☐ 2 ☐ 3 ☐ 4 ☐ 5 ☐ |
| E5 | Marriage within the same family or tribe is acceptable if both families are medically healthy. | | 1 ☐ 2 ☐ 3 ☐ 4 ☐ 5 ☐ |
| E6 | Public education about hereditary diseases should be strengthened in the Al-Baha region. | | 1 ☐ 2 ☐ 3 ☐ 4 ☐ 5 ☐ |

*All six attitude items are positively worded with respect to preventive genetic services; no reverse-coding is applied. Higher pooled scores reflect more favourable attitudes. Item wording is identical to that displayed in Figure 1 of the main manuscript.*
